# Relationship between NUDT21 mediated alternative polyadenylation process and tumor

**DOI:** 10.3389/fonc.2023.1052012

**Published:** 2023-02-02

**Authors:** Shan Xiao, Huan Gu, Li Deng, Xiongtao Yang, Dan Qiao, Xudong Zhang, Tian Zhang, Tao Yu

**Affiliations:** ^1^ Department of Oncology, Affiliated Hospital of Southwest Medical University of China, Luzhou, China; ^2^ Department of Head and Neck Surgery, Sichuan Cancer Hospital & Institute, Sichuan Cancer Center, School of Medicine, University of Electronic Science and Technology of China, Chengdu, China; ^3^ Department of Anesthesia, Sichuan Cancer Hospital & Institute, Sichuan Cancer Center, School of Medicine, University of Electronic Science and Technology of China, Chengdu, China

**Keywords:** alternative polyadenylation, microRNA, NUDT21, tumor, therapy

## Abstract

Alternative polyadenylation (APA) is a molecular process that generates diversity at the 3’ end of RNA polymerase II transcripts from over 60% of human genes. APA and microRNA regulation are both mechanisms of post-transcriptional regulation of gene expression. As a key molecular mechanism, Alternative polyadenylation often results in mRNA isoforms with the same coding sequence but different lengths of 3’ UTRs, while microRNAs regulate gene expression by binding to specific mRNA 3’ UTRs. Nudix Hydrolase 21 (NUDT21) is a crucial mediator involved in alternative polyadenylation (APA). Different studies have reported a dual role of NUDT21 in cancer (both oncogenic and tumor suppressor). The present review focuses on the functions of APA, miRNA and their interaction and roles in development of different types of tumors.NUDT21 mediated 3’ UTR-APA changes can be used to generate specific signatures that can be used as potential biomarkers in development and disease. Due to the emerging role of NUDT21 as a regulator of the aforementioned RNA processing events, modulation of NUDT21 levels may be a novel viable therapeutic approach.

## Introduction

Cancer ranks as a leading cause of death and an important barrier to increasing life expectancy in every country of the world. According to the GLOBOCAN 2020 estimates of cancer incidence and mortality produced by the International Agency for Research on Cancer, worldwide, an estimated 19.3 million new cancer cases (18.1 million excluding Non-melanoma skin cancer) and almost 10.0 million cancer deaths (9.9 million excluding Non-melanoma skin cancer) occurred in 2020 (1). The low survival rate of cancer is mainly due to the high invasion rate and easy metastasis of cancer cells in its development process, which makes it difficult to diagnose early in clinical work. Therefore, it is urgent to find out the mechanism of invasion and metastasis of cancer. The initiation and progression of cancer have long been considered as a result of the accumulation of genetic mutation, by which oncogenes are activated and/or tumor suppressor genes are inactivated. Recent studies demonstrated that alternative polyadenylation (APA) activates oncogenes without genetic mutations and facilitates cancer transformation (2). APA is emerging as a novel mechanism of gene expression regulation in normal and in disease states. Polyadenylation, a cotranscriptional process, was first identified in the nuclear extracts of calf thymus as early as the 1960s (3). Cleavage and polyadenylation are RNA maturation events that cut and add an oligonucleotide (DA) tail to the 3’ end of the nascent transcript (4, 5). This processing is to prevent mRNAs from degradation and to increase their stability. Previous studies have indicated that more than half of human genes possess multiple polyadenylation sites,called alternative polyadenylation (APA), which may produce mRNA isoforms with different protein-coding regions or 3’ UTRs of variable length (when APA occurs in the last exon) (6). However microRNAs have been shown to be a very important post-transcriptional regulator of gene expression. It can regulate gene expression by complementarily binding to recognition sequences, mostly 6-8 nt, in the 3’ untranslated regions (3’ UTR) of their target mRNAs, thus inducing mRNA degradation and/or blocking mRNA translation. Moreover, several microRNAs have been shown to be expressed abnormally in many cancer types,indicating that microRNA is closely related to carcinogenesis (7).

In the last three decades, thousands of papers have supported the existence of a class of small ncRNAs termed microRNAs (miRNAs) that have biologically relevant roles in gene regulation (8). MiRNAs are defined as short non-coding RNAs~22 nucleotides long, present in all eukaryotic cells, and highly conserved during evolution. Investigators have implicated them in many biological processes, including metabolism, cell cycle, development, differentiation, and apoptosis (9). MiRNAs contribute to both malignant and benign diseases (9). There is mounting evidence that miRNAs repress gene expression through translational repression pathways as well as through mRNA degradation (8, 10). Due to the partial complementarity to their targets, miRNAs are capable of targeting multiple genes, often in multiple sites, and some mRNAs have multiple binding sites for different miRNAs (11). It is noteworthy that Brumbaugh et al. have shown that post-transcriptional regulation plays a key role in reprogramming, transdifferentiation, and stem/progenitor cell differentiation.NUDT21 is a highly conserved hydrolase, which constitutes a subunit of the CFIm complex required for 3’ RNA splicing and polyadenylation.Mechanistically, NUDT21 suppression exerts its effect on cell fate by inducing a widespread switch of APA patterns in over 1,500 transcripts (12). At present, studies have shown that NUDT21 is involved in the occurrence and development of many kinds of tumors, including glioblcomplementarily astoma (13), cervical cancer (14), breast cancer (15, 16), bladder cancer (17), pancreatic ductal adenocarcinoma (18), small cell lung cancer (19), gastric tumorigenesis (20). The lack of understanding of the mechanisms and regulation of APA, miRNA and their interaction and roles has underscored the need for further research regarding their role in cancer and other diseases.This paper will describe the impact on tumor from the relationship between miRNA, APA and NUDT21.

## APA process of gene expression

Each segment of gene expression with genetic information includes two steps: transcription and translation, that is, a DNA strand is used as a template for transcription into mRNA, and mature mRNA is translated into protein or other products. In the process of transcription, when a gene is transcribed into a precursor mRNA by RNA polymerase II, it has to go through multiple processing steps to become a mature mRNA, which is the key step of transcription. The formation of mature mRNA mainly involves 5’ terminal capping, splicing and 3’ terminal polyadenylation (21). Most eukaryotic mRNA precursors (premRNAs) must undergo extensive processing, including cleavage and polyadenylation at the 3’-end. Processing at the 3’-end is controlled by sequence elements in the pre-mRNA (cis elements) as well as protein factors (22). The 3’-end processing machinery also has important roles in transcription and splicing. Non coding RNA (ncRNA) is usually not translated into protein RNA, including miRNA, lncRNA and circle RNA (23), which can regulate mRNA expression at the transcriptional and post transcriptional levels (24).

MicroRNAs (miRNAs) are a group of highly conserved small non-coding RNAs, with a vital role in regulating the expression of protein coding genes, and could function either as oncogenes (oncomiRs) or tumor suppressors. MiRNAs are key players participating in different stages of the signal transduction process (5). They function by targeting several mRNAs affecting a multitude of transcripts to control cellular metabolisms; therefore, their dysregulation influences numerous cancer relevant processes such as proliferation, differentiation, apoptosis, and metastasis (25). They function to stabilize mRNA transcripts *via* post-transcriptional gene silencing through inhibiting the translational process of their target mRNAs *via* binding partially or fully to complementary sequences of mRNA 3′-untranslated region (3′ UTR) (26). Owing to the genomic events including mutations, deletions, amplifications, or transcriptional changes, miRNAs are dysregulated in several diseases including cancer (27). In the processing of the 3’ termini, the 3’ end of nascent mRNA is cleaved, followed by addition of a poly(A) tail (i.e., polyadenylation) (28). Both cleavage and polyadenylation occur at polyadenylation sites (PASs) which are located within the 3’ untranslated regions (3’ UTRs), introns, or internal exons (29, 30). Most eukaryotic genes contain multiple PASs. A conserved hexameric sequence AAUAAA (31), occurring upstream of the PASs, contains the most important signal (i.e., poly(A) signal) of pre-mRNA cleavage and polyadenylation. Both this canonical poly(A) signal and the PASs are widespread in eukaryotic mRNA. According to the analysis, the status of 3’ UTRs do not affect their own RNA level. Instead, they significantly influence their binding RBP’s downstream targets. However, it is still possible that the changed 3’ UTRs might affect their protein translation even though they not affect mRNA level directly (32). Alternative poly(A) sites can be located in the last or 3’-most exon, giving rise to mRNAs with variable 3’-untranslated region (3’-UTR), or in a different exon, resulting in protein products that vary at the C-terminus (33). Polyadenylation of eukaryotic mRNA occurs in a step-wise process, which includes a specific cleavage at the 3’ end of nascent mRNA followed by an addition of a poly(A) tail. Six factors, namely CPSF, CstF, CFIm, CFIIm (cleavage factor II), PAP (poly(A) polymerase) and PABII (poly(A) binding protein II) have been characterized to facilitate the 3’ end processing (34, 35). The cleavage reaction requires recognition by CPSF of AAUAAA hexamer upstream of the cleavage site and by CstF of degenerate GU- and U-rich sequences downstream of the cleavage site. CFIm is an essential pre-mRNA 3’ end processing factor unique to metazoans, which facilitates assembly of 3’ end processing factors on pre-mRNA *in vitro* (36). 3′ UTRs contain regulatory element binding sites of miRNA and RNA binding protein, which allow the regulation of these genes and provide an important gene expression regulatory layer. Different lengths of 3’ UTR can lead to the inclusion or deletion of cis regulatory elements (miRNA and RNA binding protein) binding sites, thus affecting the stability, transport and translation efficiency of mRNA. It is worth noting that some fast-growing cell populations (including cancer cells) prefer to generate mRNA with shorter 3’ UTR by APA process, while static or differentiated cell populations tend to generate mRNA with longer 3’ UTR (37, 38). Studies have shown that the number of proteins translated by an mRNA depends on its 3’ UTR length, and shorter 3’ UTR transcripts can produce higher protein levels. When APA produces the same protein, the production of protein will also be affected by the increase or decrease of regulatory elements in 3’ UTR. In a word, APA promotes the complexity of RNA structure and diversity of RNA function, which complicates the transmission of genetic information from genome to phenotype Group (39, 40). The diverse 3′ UTRs generated by APA may confer different stability, translation efficiency, or subcellular localization to the mRNA isoforms (41).

## miRNA, APA and cancer

At present, many studies have found that polyadenylation is an RNA-processing mechanism that generates distinct 3’-termini on messenger RNAs, producing messenger RNA isoforms. Different factors influence the initiation and development of this process. All forms of APA (both splicing-APA and tandem UTR-APA) involve changing the position of the poly(A) tail making the identity of the 3′-UTR different between each mRNA isoform (42). The majority of the 3′-UTR changes are quite likely to be impactful because an estimated 70% of protein-coding genes in humans have conserved microRNA (miRNA) target sites and ~11% have AU-rich elements (AREs) within their 3′-UTRs (43–45). Over the past decade, significant attention has been focused on the role that 3′-UTR localized microRNA-binding sites and AREs play in modulating gene expression of protein-coding genes (46, 47). Up to now, at the genomic level, a number of single-nucleotide polymorphisms have been identified that disrupt miRNA-binding sites in the 3′-UTRs of genes associated with increased cancer risk and poor survival (48). As we know, 3′-end polyadenylation is a critical step of eukaryotic mRNA processing to maturation (49). Alternative polyadenylation (APA) generates multiple mRNA isoforms, among which the shorter ones can escape from translation repression or mRNA degradation mediated by microRNAs (miRNAs) or other RNA regulatory elements within its 3′-UTRs (50). Recently, shortening of mRNA 3′-UTRs has been reported to be involved in the pathogenesis and progression of certain malignancies (39, 44, 46). A prevailing hypothesis is that it induces proto-oncogene expression in cis through escaping microRNA-mediated repression (51). Their results suggest a major role of 3’ UTR shortening in repressing tumor-suppressor genes in trans by disrupting ceRNA crosstalk, rather than inducing proto-oncogenes in cis (51, 52).

1. The role of APA as RNA regulation process has been reported in various human physiological conditions and diseases. Transcripts with longer 3′ UTRs were observed during embryonic development (53) and neuron differentiation (54), as well as the development of the central nervous system (55, 56). Several recent studies have shown that global 3′ UTR shortening is present in malignancies (39, 57). The global 3’UTR shortening landscape and 3’UTR shortening of specific genes could have opposite effect thus when we discuss about the impact of Nudt21 on tumor growth it should be evaluated on a case by case basis. In addition, APA plays an important role in cellular processes, including cell proliferation (58, 59), cell fate determination (12). The consequences of APA can be significant, with effects on post-transcriptional gene regulation, including mRNA stability, translation, nuclear export, and cellular localization (60). Many studies have found that APA process is closely related to the occurrence and development of tumors. As mentioned earlier, Rehfeld et al. used high-throughput sequencing data to map poly(A) sites and characterize polyadenylation genome-wide in three small intestinal neuroendocrine tumors(SI-NETs) and a reference sample. In the tumors, 16 genes showed significant changes of APA pattern, which lead to either the 3′ truncation of mRNA coding regions or 3′ untranslated regions. Among these, 11 genes had been previously associated with cancer, with 4 genes being known tumor suppressors: DCC, PDZD2, MAGI1, and DACT2 (61). Li et al. in 2020 identified that the 3′-UTR shortening of fibronectin type III domain containing 3B (FNDC3B) mRNA mediated its overexpression in Nasopharyngeal carcinoma(NPC)and promoted NPC progression by targeting myosin heavy chain 9 (MYH9) (62). This newly identified FNDC3B-MYH9-Wnt/β-catenin axis could represent potential targets for individualized treatment in NPC. Meanwhile, another study showed that the use of tandem APA sites was prevalent in NPC, and numerous genes with APA-switching events were discovered (63) In total, they identified 195 genes with significant differences in the tandem 3′ UTR length between NPC and normal nasopharyngeal epithelial tissue (NNET): including 119 genes switching to distal poly (A) sites and 76 genes switching to proximal poly (A) sites. Several gene ontology (GO) terms were enriched in the list of genes with switched APA sites, including regulation of cell migration, macromolecule catabolic process, protein catabolic process, proteolysis, small conjugating protein ligase activity, and ubiquitin-protein ligase activity. Lai et al. found that the new mRNA isoforms in MKN28 cell line contained shorter 3’ UTR compared with MKN45 and AGS cell lines (64). MKN28 is a kind of gastric adenocarcinoma with lymph node metastasis, which is more malignant than the other two gastric cancer cell lines. This result suggests that the new isoforms containing shorter 3’ UTR mRNA may be closely related to the malignant degree of the tumor. Similarly, the 3’ UTR of SEC11A gene was significantly shorter than that of normal cells, but it was previously reported that SEC11A gene was overexpressed in gastric cancer (65). Fu et al. found that the shortening of 3’ UTR caused the deletion of miRNA recognition sites, resulting in gene overexpression (66). Therefore, the overexpression of SEC11A in gastric cancer cells may be related to the emerging APA sites. In 2021, Venkat et al. reported widespread, recurrent, and functionally relevant 3′-UTR alterations associated with gene expression changes of known and newly identified pancreatic ductal adenocarcinoma (PDAC) growth-promoting genes and experimentally validate the effects of these APA events on protein expression (67). They found enrichment for APA events in genes associated with known PDAC pathways, loss of tumor-suppressive miRNA binding sites, and increased heterogeneity in 3′-UTR forms of metabolic genes. According to the researches, Ki-67 is an independent prognostic factor in early breast cancer (68) and in neoadjuvant therapy (69). Yan et al. identified a novel post-transcriptional mechanism, involving APA and miRNA, that underlies the elevated expression of Ki-67 in breast cancer (70). The results have shown that breast cancer cells preferentially express Ki-67 mRNA isoforms with short 3’UTRs, and the expression of shorter Ki-67 mRNAs leads to an increase in Ki-67 mRNA stability and translational efficiency. In all, Ki-67 promotes the proliferation and migration of breast cancer by replacing the interaction between polyadenylation (APA) and microRNA. It has been known that the expression ratio between long isoforms and short isoforms (LSR) of overall genes is variable across different tissues or cell types (71, 72). Interestingly, Sandberg et al. found that the ratio has a strong negative correlation with cell proliferative state (50). In addition, cancer cells exhibit significantly lower ratios than normal tissues and untransformed cells (46). Liaw et al. retrieved public APA annotations and isoform expression profiles of breast cancer and normal cells from a high-throughput sequencing method study specific for the mRNA 3′end (73). Combining microRNA expression profiles, they performed statistical analysis to reveal and estimate microRNA regulation on APA patterns in a global scale. They found that: 1). The amount of microRNA target sites in the alternative UTR (aUTR), the region only present in long isoforms, could affect the LSR of the target genes; 2). The genes whose aUTRs were targeted by up-regulated microRNAs in cancer cells had an overall lower LSR; 3). The target sites of up-regulated microRNAs tended to appear in aUTRs. Finally, they demonstrated that the amount of target sites for up-regulated microRNAs in aUTRs correlated with the LSR change between cancer and normal cells. The results indicate that up-regulation of microRNAs might cause lower LSRs of target genes in cancer cells through degradation of their long isoforms. Singh et al. suggested that alternative mRNA processing, particularly APA, can be a powerful molecular biomarker with prognostic potential (74). By the global shortening of 3’ UTRs *in vitro* and *in vivo*,the 3’ UTRs show distinct features in primary cancer samples (75). With shortened 3’ UTRs, functional consequences have been produced by genes, which has led to greater mRNA stability and increased protein output (46). Through forcing the expression of shorter 3’ UTR isoforms, phenotypic consequences were observed, which suggests that 3’ UTR shortening is associated with cell proliferation, including T-cell activation or early embryogenesis (50, 53). WANG et al. found a significantly greater number of genes with shortened 3’ UTRs in the samples with luminal B breast cancer (76). According to the research, there are 64 significant differentially expressed miRNAs (DERs) in colorectal cancer (CRC) patients (77). Their target genes were related to cell adhesion and transcription regulation and were prevailingly involved in the CRC-related pathway. Integrative analysis of the miRNA and APA profile revealed 16 DERs were correlated with 12 polyadenylation factors, and six of them were significantly differently expressed in CRC. Meanwhile they also found four DERs that lost binding sites due to APA and showed a positive correlation between the miRNA and gene expression. The above data show that miRNAs regulated APA by modulating key polyadenylation factors, and several miRNAs lost their suppression on mRNA due to APA. The current study performed a systematic identification and analysis of survival-associated genes from the perspectives of AS, APA and DNA methylation in gynecology cancers (78). The results indicate that the mRNA expression levels of CIRBP and INPP5K are significantly downregulated and associated with the clinical outcomes of patients with cervical squamous cell carcinoma and endocervical adenocarcinoma (CESC), uterine corpus endometrial carcinoma (UCEC) and ovarian serous cystadenocarcinoma (OV). Further enrichment analysis confirmed that these prognostic genes possibly modulate cancer progression by inducing T cell activation. Similarly, it has been identified that remarkably consistent alternative 3’ UTR isoforms between the two cohorts, most of which were shortened in lung adenocarcinoma (LUAD) (79). The research further suggested that aberrant usage of proximal poly(A) sites resulted in escape from miRNA binding, thus increasing gene expression. Notably, it also showed that the 3’ UTR lengths of the mRNA transcriptome were correlated with the expression levels of APA factors. These results suggest that APA events, both 3′ -UTR shortening and lengthening, play important roles in cancer etiology and treatments.

## NUDT21 and APA and tumor

NUDT21 (also known as CPSF5 and CFIm25) is a highly conserved hydrolase, which constitutes a subunit of the CFIm complex required for 3’ RNA splicing and polyadenylation (80). Studies have found that silencing NUDT21 can lead to changes in the utilization of poly(A) sites in other genes, thus promoting the selection of proximal poly(A) sites and ultimately shortening the 3’ UTRs of cell mRNA (81, 82). Analysis of NUDT21 target showed that 3’ UTRs of corresponding mRNA enriched multiple miRNA binding sites. When NUDT21 was knocked out, the Poly(A) sites in JMJDC1, RYBP and WDR5 target genes shifted from the far end to the near end, resulting in the loss of miR-34C and miR-29A binding target sequences. This finding confirmed that escaping from miRNA mediated degradation is an important mechanism of NUDT21 silencing increasing cell reprogramming. NUDT21 posttranscriptional regulation of APA plays a key role in gene reprogramming, stem cell directed differentiation and tumor progression (12). NUDT21, also referred to as cleavage and polyadenylation specificity factor subunit 5 (CPSF5), is a subunit of the cleavage factor Im (CFIm) complex required for 3′-UTR cleavage and polyadenylation. Recently, the mammalian cleavage factor I 25-kD subunit (CFIm25; encoded by the gene Nudt21) was discovered as a master regulator of APA among 15 cleavage and polyadenylation factors (81, 83). NUDT 21 is responsible for sequence-specific recognition of the element UGUA upstream of the poly (A) site (PAS), and thus plays a determinant role in APA (84, 85). In mammals, the factors that are required for mRNA maturation *in vitro* include the cleavage and polyadenylation specificity factor (CPSF), cleavage stimulatory factor (CstF), cleavage factors Im and IIm (CFIm and CFIIm), poly(A) polymerase (PAP) and nuclear poly(A) binding protein (PABN2). The cleavage reaction requires CPSF, CstF, CFIm, CFIIm, and PAP. CPSF binds the highly conserved AAUAAA hexamer upstream of the cleavage site, and CstF binds the GU/U-rich sequence downstream of the cleavage site (86). CFIm25 can regulate the selection of poly(A) site in this way, promote the assembly of pre-mRNA 3′-end processing complex, and improve the splicing rate and efficiency of polyadenylate site *in vitro* (87). As a key factor in alternative polyadenylation, cleavage factor I m25 plays an important role in messenger RNA maturation and cell signal transduction. Moreover, by regulating the process of alternative polyadenylation, it can inhibit the proliferation, invasion, and metastasis of a variety of tumors. Sun X et al. discovered cleavage factor I m25 also acts as an oncogene in select tumors (88). Recently, a unbiased shRNA screen revealed that NUDT21 expression is a major barrier for creation of iPS cells from fibroblasts (12). In this context, downregulation of NUDT21 will induce broad shortening of 3′-UTRs enhancing differentiation of cells leading to a stem-like state (12). It has been shown that CFIm25 stimulates the use of PASs that are enriched in UGUA elements, which are most prevalent at distal PASs (dPAS) (12). Research found that depletion of NUDT21 not only causes increased pPAS usage but also increases cell proliferation and enhances glioblastoma(GBM) cell tumorigenicity (83, 89). Mechanistically, NUDT21 suppression exerts its effect on cell fate by inducing a widespread switch of APA patterns in over 1,500 transcripts (12). Inhibition of NUDT21 can enhance the induction of pluripotent stem cells, promote the transformation of trophoblast stem cells, and affect the differentiation of myeloid precursor and embryonic stem cells, indicating that NUDT21 plays a broad role in the process of cell differentiation. APA of the poly (A) splice site at the 3′-end of the mRNA precursor was carried out in different environments to produce mature mRNA with different 3′ untranslated region (3′ UTR) or different coding ability. Different lengths of 3′ UTR can cause changes of RNA binding protein or microRNA binding site, and then affect the localization, stability and translation of mRNA. Related studies have shown that NUDT21 can guide APA to regulate 3′ UTR length by combining proximal cleavage and polyadenylation sites (90). NUDT21-mediated alternative polyadenylation has been shown to influence various cell fate decision processes as well as tumorigenesis (12, 83). In particular, depletion of NUDT21 leads to global 3′ UTR shortening, which has been linked to increased tumorigenesis and tumor development (91). Saeko et al. demonstrated the cytoplasmic localization of NUDT21 and its unexpected role in regulation of antiviral proteins in the cytoplasm in addition to its well-established localization to nucleus and function in alternative polyadenylation (92). According to the research, Masamha et al. demonstrated that downregulation of NUDT21 enhanced the tumorigenic properties and increased the tumor size, whereas the upregulation of NUDT21 inhibited the growth and tumorigenicity of GBM cells (83). In hepatocellular carcinoma, Sun et al. showed that downregulation of NUDT21 led to enhanced cancer cell proliferation *via* shortening the 3′ UTR of several oncogenes (93). Meanwhile, NUDT21 can function as an oncogene under certain conditions. In 2020, Xing et al. found that NUDT21 was remarkably downregulated in Cervical Cancer(CxCa) tissues and cells, and that the aggressive phenotype of CxCa cells, including proliferation, migration, and invasion were inhibited by NUDT21 overexpression; conversely, silencing NUDT21 yielded the opposite effect (14). 3′-UTR shortening (3′ US) was reported widespread in diverse types of human cancers (39). Using PRIMATA-APA on the cancer genome atlas (TCGA) breast cancer data, Kim et al. found that the global APA events collectively increase or decrease the target sites of the miRNAs that are known to regulate cancer etiology and treatments (15). Their results were replicated in a reanalysis of NUDT21 knockdown (KD) data. NUDT21 knockdown (KD) was shown to induce global 3′ US events and promote tumorigenesis *in vivo* and *in vitro* by removing miRNA target sites to repress tumor suppressor genes (83). They also found that although the NUDT21 KD in HeLa Cells and the TCGA breast cancer carry a distinct set of 3′ US genes, they change the target sites of the common miRNAs (tamoMiRNA), suggesting that the APA initiated tumorigenesis is attributable to the miRNA target site changes, not the APA events themselves (51). Weng et al. demonstrated a consistent downregulation of CFIm25 in skin samples collected from systemic sclerosis patients, and this downregulation was mainly detected in (myo)fibroblasts (94). RNA-seq detected significant APA events in human skin fibroblasts upon CFIm25 KD and identified important fibrotic TGFβ-regulated genes targeted by CFIm25. Previous studies have shown that the production of a eukaryotic protein-coding messenger RNA (mRNA) requires the recognition of a specific poly(A) site sequence at the end of the gene. The deletion of CFIm25 or CFIm68 promotes the use of a proximal poly(A) site, thereby affecting the function of the 3′ untranslated region (3′UTR) of many mRNAs (95). In addition, Elkon et al. suggested that when located in the last or 3′-most exons, alternative poly(A) sites could lead to the production of mRNAs with variable 3′-untranslated regions, resulting in protein products that vary at the C-terminus (96). It suggests that CFIm generally promotes the recognition of distal poly(A) sites. Related studies have shown that the misregulation of CFIm is associated with the tumorigenicity of glioblastoma and some neuropsychiatric diseases by altering the length of the 3′ UTR of mRNA (83). In 2020, Wang et al. found that NUDT21 might play a tumor-suppressive role by inhibiting cell proliferation and invasion *via* the NUDT21/CPSF6 signaling pathway in breast cancer cells (16). In HeLa Cells, it was found that the most significant poly (A) site transition was found after NUDT21 knockout, and abnormal expression of several oncogenes including cyclin D1 (83). Recently, Chu et al. have identified that NUDT21 regulates the APA of a broad spectrum of mRNA in GBM, with target genes enriched in the Ras signaling pathway (13). They also find the activated oncogenic function of Pak1 is potentiated by NUDT21-regulated 3′-UTR shortening and therefore can contribute to GBM development and progression. Further, their results reveal that both NUDT21 and Pak1 may serve as a biomarker for predicting prognosis of GBM patients and imply an important role in GBM development and progression. Moreover, NUDT21 expression is reduced in low-grade glioma(LGG) grade II and grade III, and all four GBM subtypes relative to normal brain tissue. Collectively, these results suggest that reduction in NUDT21 is an important component of GBM tumor progression. The research in 2019 for the first time provide novel insight into the crucial role played by NUDT21 in regulation of APA-mediated 3′-UTR alterations, which further promote bladder cancer (BC) progression (17). Here they reported NUDT21 inhibits cell proliferation, migration, and invasion, and represses tumorigenicity in BC. It characterized NUDT21-regulated genes with shortened 3′-UTRs, and found that ANXA2 and LIMK2 contribute to NUDT21-mediated tumor suppression by augmenting Wnt and NF-κB signaling. Recently, it has been reported that loss of NUDT21 increased usage of proximal polyadenylation sites and produced shorter 3′ UTR in various oncogenes, such as PSMB2 and CXXC5, which had fewer miRNA binding sites, escaped from miRNA-mediated gene repression, and further promoted hepatocellular cancer(HCC)cell proliferation and invasion (2, 93). In addition, Sun et al. reported that loss of NUDT21 shortens the 3′-UTR of various oncogenes (mainly RAB3IP, TMEM267, UBA5, and CCT5) in HCC cells, leading to unregulated tumor cell proliferation (93). In hematologic malignancy, silencing NUDT21 inhibits proliferation and promotes apoptosis of human K562 leukemia cells through regulation of p-ERK expression (97). Similarly, In Huang’s study, they found KD of CFIm25 promoted its 3′ UTR shortening (98). The shortened transcripts were sufficient to increase its protein turnover without increasing its transcript level, suggesting that 3′ UTR shortening of IGF1R could be a mechanisms that promotes its protein expression in Non-small cell lung cancer(NSCLC). This mechanism was further confirmed by RNA-seq data from the Cancer Genome Atlas which showed a significant 3′ UTR shortening of IGF1R in lung cancer samples compared to normal donors.Traditionally, CFIm25 is known to facilitate 3’ end formation of pre-mRNAs resulting in the formation of polyadenylated transcripts. Recent studies suggest that CFIm25 may be involved in the cyclization and hence generation of circular RNAs (circRNAs) that contain UGUA motifs (99). These circRNAs act as competing endogenous RNAs (ceRNAs) that disrupt the ceRNA-miRNA-mRNA axis. Different studies have reported a dual role of CFIm25 in cancer (both oncogenic and tumor suppressor). microRNAs (miRNAs) may be involved in CFIm25 function as well as competing endogenous RNAs (ceRNAs) (100). Last year Zhu et al. published that NUDT21 promoted cell proliferation, colony formation, cell migration and invasion through modulating SGPP2 in human gastric cancer (101). They found that NUDT21 expression was positively correlated with tumor size, lymph node metastasis and clinical stage in gastric cancer patients. High level of NUDT21 was associated with poor overall survival (OS) rates in gastric cancer patients. A recent work published by Witkowski et al. on Nature Immunology also reported the negative role of Nudt21 in chimeric antigen receptor T cell therapy (CAR-T) (102). They foung that NUDT21 limits CD19 levels through alternative mRNA polyad xsenylation in B cell acute lymphoblastic leukemia.Nudt21 has been reported to both prohibit and promote tumor progression.In many cancers, NUDT21 acts as a tumor suppressor. However, there are some cancers where NUDT21 has the opposite effect. These studies indicate the mechanism underlying NUDT21-mediated tumor suppression may be cancer specific and have different features in different malignancies. Because of the key role of APA in mRNA output, we hypothesized whether NUDT21 could affect the occurrence and development of oral cancer by controlling the APA process.

## Conclusion

Revealing the molecular mechanism of malignant tumor pathogenesis and progression may help to develop new and effective early detection, diagnosis and prediction strategies. The occurrence of cancer is a multifactorial process, involving many genetic and epigenetic processes, which can change the functions of tumor suppressor genes, oncogenes and other related molecules. Malignant tumors have difficulty in early diagnosis, high metastasis rate and poor prognosis. In recent years, the search for tumor related epigenetic biomarkers has shown an upward trend, especially the study of miRNA and APA. Herein above statement, we need to find novel regulatory molecules and epigenetic biomarkers. With the research of tumor, more and more tumor related genes and molecular mechanisms have been gradually explored, such as NUDT21 and APA. Up to date, it has been reported that the APA process mediated by NUDT21 is closely related to the biological behavior of many tumors. Due to the lack of current knowledge regarding the mechanisms of action and regulation of NUDT21 and alternative polyadenylation, it is necessary to further examine their role in cancer as well as in other diseases. This provides a new idea for us to study tumor. Studying the expression of NUDT21 mediated APA process in cancers may become an attractive field, which will bring new markers for cancer diagnosis, prognosis and new therapeutic targets. The significance of NUDT21 and APA in tumorigenesis, metastasis and progression needs to be studied. They may be important regulatory molecules in the occurrence and development of tumors. The discovery of this epigenetic biomarker may have an impact on the early diagnosis and prognosis of tumor patients.

## Author contributions

SX: Data,Writing- Original draft preparation, Validation, Content management and manuscript revision. HG: Conceptualization, Methodology, Software. LD: Revision of the manuscript process, made a contribution. XY, DQ: Visualization, Investigation. XZ: Check and modify. TZ: Inspection and testing. TY: Writing- Reviewing and Editing. All authors contributed to the article and approved the submitted version.
